# Healthcare professionals’ perceived problems in fast-track hip and knee arthroplasty: results of a qualitative interview study

**DOI:** 10.1186/s13018-019-1334-3

**Published:** 2019-09-04

**Authors:** Miia Marika Jansson, Marja Harjumaa, Ari-Pekka Puhto, Minna Pikkarainen

**Affiliations:** 1 Research Group of Medical Imaging, Physics and Technology, University of Oulu, Oulu, Finland; 2 VTT Technical Research Centre of Finland, P.O.Box 1100, FI-90571 Oulu, Finland; 3 Division of Operative Care, Department of Orthopaedic and Trauma Surgery, Oulu University Hospital, P.O.Box 21, FI-90029 Oulu, Finland; 4 Martti Ahtisaari Institute, Oulu Business School, Oulu University, P.O.Box 8000, FI-90014 Oulo, Finland

**Keywords:** Arthroplasty, New information and communication technologies, Patient journey, Qualitative research

## Abstract

**Background:**

Fast-track and outpatient arthroplasty methodologies combine evidence-based clinical features with organizational optimization resulting in a streamlined pathway from admission to discharge and beyond. This qualitative study explored perceived problems of healthcare professionals during fast-track hip and knee arthroplasty.

**Methods:**

Semi-structured interviews were conducted with four surgeons, two anesthesiologists, ten nurses, and four physiotherapists. An inductive content analysis was used to analyze the data. NVivo qualitative data analysis software was used.

**Results:**

Analysis of the data revealed eight main categories of problems: patient selection, referrals, meeting the Health Care Guarantee, patient flow, homecare, patient counseling, transparency of the journey, and receiving feedback. In addition, problems related to information flows and communication, responsibilities between different stakeholders, and existing information systems were identified.

**Conclusions:**

The study revealed that healthcare professionals perceived several problems during the fast-track journey that reduce its effectiveness and make it more difficult to meet the Health Care Guarantee. Problems could be alleviated by changing internal and external organizational practices, as well as by developing new information and communication technologies that would provide up-to-date communication channels for healthcare professionals and patients. In addition, new collaboration mechanisms should be developed in order to solve the problems that occur across different organizations.

## Introduction

Refinements of surgical techniques and devices, anesthesia protocols, and patient selection have facilitated fast-track [1] and outpatient arthroplasty [2, 3] while the advantages of these methodologies are well-documented from both the socio-economic [4, 5] and patient perspectives [6]. Fast-track and outpatient arthroplasty methodologies have combined evidence-based clinical procedures with organizational optimization resulting in a streamlined pathway from admission to discharge and beyond [3, 6].

In the past decade, the number of total hip arthroplasty (THA) and total knee arthroplasty (TKA) operations has increased threefold while staffing levels have remained unchanged [7]. At the same time, access to healthcare services has been a critical issue while long waiting times for core specialized healthcare services have been consistently identified as a key barrier to access to care [8]; according to the Finnish Primary Health Care Act (66/1972), a healthcare professional must evaluate the patient’s need for treatment within 3 weeks of the hospital receiving patient’s referral. If a healthcare professional estimates that treatment is necessary, treatment must begin within 6 months according to the Health Care Act. The need for focused, proactive care to improve outcomes and avoid unnecessary hospital days is urgent.

In Finland, pressure on health budgets, reductions in hospital beds, and patient expectations have changed the nature of nursing care and there is currently more emphasis on communication in order to develop the patients’ capacity for self-care at home [7, 9]. At the same time, patients have been more motivated to play an active role in their own treatment, care, and rehabilitation. For instance, the usage of the Internet has increased rapidly: 79% of patients had access to the Internet in 2012, and among them, 23% in 2010 to 65% in 2012 had used the internet to research their orthopedic conditions or upcoming treatment [10, 11]. In addition, telephone-delivered interventions in medical consultancy and communication between patients and medical specialists have tripled during the 2014–2017 period [12].

For patients with THA/TKA, novel information and communication technologies have been more effective than standard inpatient care in improving patient satisfaction [13–17] and physical functioning [13, 18], as well as for promoting planning and self-efficacy [14] and reducing resource utilization [17, 19, 20] without an increase in adverse events. In this study, we aimed to explore perceived problems of healthcare professionals during the fast-track THA/TKA journey to inform the future design and delivery of healthcare for patients undergoing joint replacements. In this study, the patient journey refers to the steps that the patient goes through in a course of treatment, regardless of the planned clinical pathway for his or her status. Improvements to the patient journey aim to maximize the patient experience, not only the efficacy and efficiency of the treatment.

## Material and methods

### Study design

A qualitative cross-sectional interview study, approved by the Ethics Committee of the Northern Ostrobothnia Hospital District (Decision No: 83/2018), was used to explore healthcare professionals’ perceived problems during the fast-track THA/TKA journey to inform the future design and delivery of healthcare for patients undergoing joint replacements. This work is an exploratory case study with an interpretative nature. Reporting of the study was performed in accordance with the consolidated criteria for reporting qualitative research [21].

### Setting and participants

This study was conducted within a single joint replacement center in a 900-bed, tertiary-level, university teaching hospital in Finland, serving an area comprising 735,000 inhabitants. During the study period, multidisciplinary preoperative surgical visits in conjunction with patient education, mobilization on the day of the surgery, and well-defined discharge criteria were standard procedures in the treatment of patients undergoing THA and TKA procedures [1]. Participants were purposively selected using maximum variation sampling [22] and recruited by the corresponding author. The participants were eligible if they (1) were willing and able to give informed consent for participation in the study; (2) were able to speak, read, and understand Finnish; (3) were an employee of the hospital; (4) provided care for joint replacement surgery patients; and (5) owned a smartphone or tablet computer. The concept of information power was utilized to determine an adequate sample size [23]. Two participants dropped out before completing the interviews due to sudden sickness, but otherwise there were no refusals.

### Data collection

**Fig. 1:**
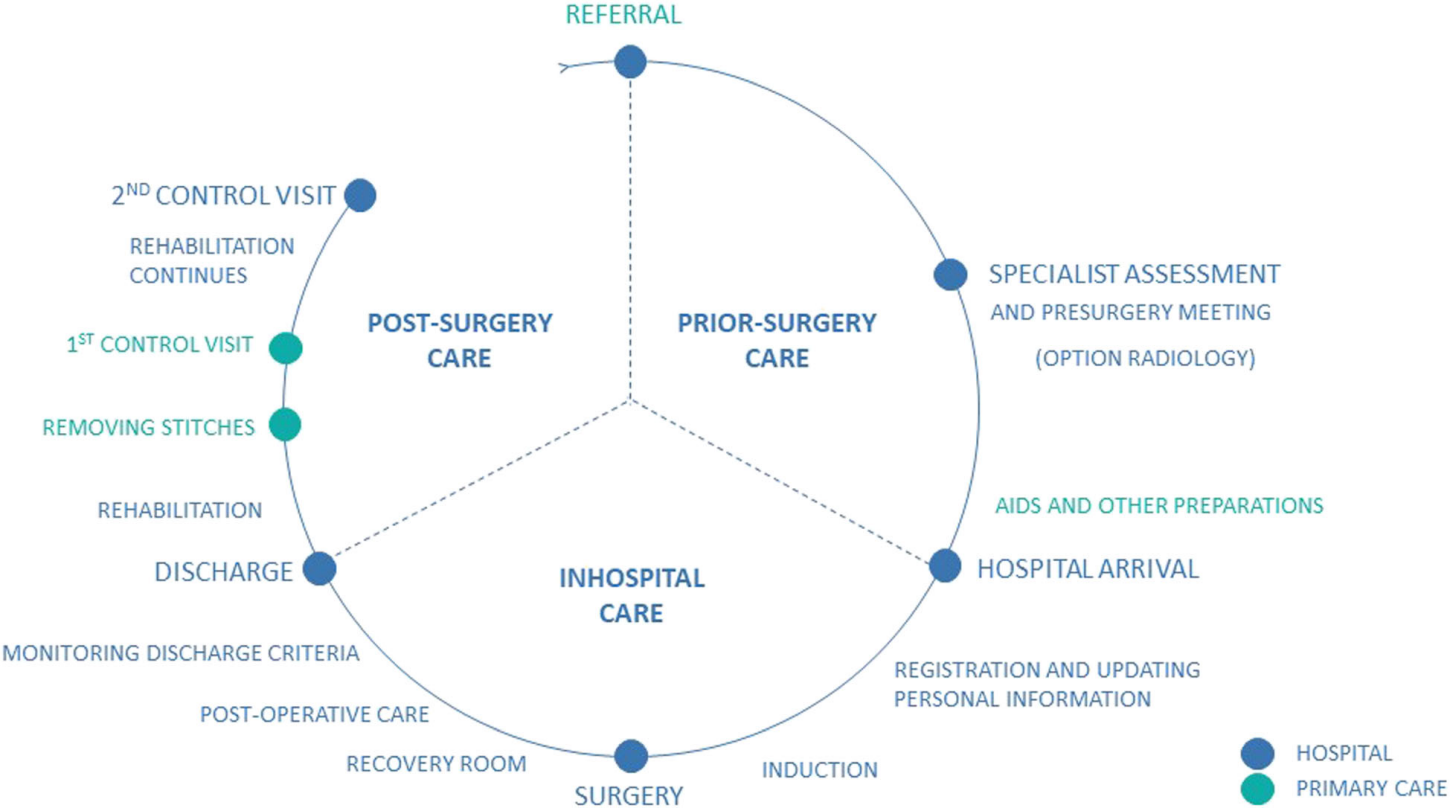
An overview of current patient journey. We distinguished four clearly separated phases that the patients go through which may involve several events: (1) pre-referral primary care (long, often uncertain process with pain), (2) the specialist assessment and preoperative surgical visit (all patients receive oral counseling in conjunction with written material about their surgery and preliminary home care instructions), (3) in-hospital care (patients undergo surgery and spend approximately 40–64 h on the ward. Patients are discharged with written post-surgery home care instructions about wound care, removal of stitches, analgesia, physical activity, potential complications, and instructions for follow-up), and (4) homecare (the first control visit will be in primary care in 2 weeks post-discharge. The second control visit will be in hospital at 6–8 weeks post-discharge for patients with knee replacements and in 8–12 weeks for patients with hip replacements. Patients do not meet their surgeon unless they have complications)

At the outset of the project, process mapping was used as a technique to build a comprehensive understanding of the current fast*-*track journey (Fig. 1). As a result of the process mapping, a patient journey from primary care to the control visit at the hospital was formed in cooperation with healthcare professionals [24]. The journey was partly based on previous work done in lean transformation projects.

The period for recruitment and data collection lasted from the 8th of December until the 7th of November 2018. Prior to the interview, the corresponding author introduced herself (e.g., name, occupation, affiliations) and explained the purpose of the research and that the interview would be audio-recorded and transcribed and the collected data would be pseudonymized. Those who agreed to participate and to the interviews being audio-recorded signed a consent form prior to the interview. Participation was voluntary, and the participants could withdraw without giving a reason for their withdrawal.

Private, face-to-face interviews were carried out in an undisturbed room at the hospital by the corresponding author (PhD), who was experienced in conducting qualitative studies in the acute and critical care settings. The corresponding author did not have a direct working relationship with the participants. The interviews were semi-structured, and notes were taken. A written topic guide, based on the process mapping, was used (Table 1). Open questions, such as “What challenges have you encountered in scheduling?” and “What challenges have you encountered in the fulfillment of discharge criteria in the target time?,” were asked. Due to the explorative design, the participants had the opportunity to bring up the issues that were most important to them.

**Table 1:** Semi-structured topic guide

Themes	
1. Demographics	
2. Gaming behavior and orientation	
3. Perceived problems prior to, during, and post-surgery	
a. Relevant information	
b. Most frequently asked questions	
c. Evaluation of eligibility	
d. Patient counseling	
e. Adverse events	
f. Fulfillment of discharge criteria in target time	
4. Perceived problems related to general process	
a. Challenges in the current journey	
b. Transparency of journey	
c. Feedback prior to, during, and post-surgery	

The interviews lasted between 22 and 58 min (mean 41.2 min). The adequacy of the final sample size was evaluated continuously throughout the interviews [23]. The information power achieved demonstrated a sufficient sample size for the study. All the data was treated as confidential and transcribed immediately by a transcription service provider. The physical data was stored under lock and key at the university, and the digital data was stored on the research organizations’ professionally maintained servers protected by password. All the researchers processing the raw interview data signed a data processing agreement.

### Data analysis and rigor

Data from the transcribed interviews was analyzed by the means of an inductive content analysis [25]. The software package NVivo 12 Plus for Windows was used for coding, grouping, categorization, and abstraction. Firstly, all the answers were collected in sub-categories based on the respondents’ descriptions (for instance capacity) using open coding. Secondly, similar open codes were grouped together into a generic category (for instance resources) and a main category (for instance, problems to meet the Health Care Guarantee) and labeled using content-specific keywords. The abstraction process continued as far as it was reasonable and possible through manual and digital tabulation. Finally, the open codes were quantified within each generic category.

Rigor was demonstrated ensuring credibility, dependability, conformability, and transferability [26]. To achieve credibility, the interviews were audio-recorded and transcribed verbatim to ensure that all the responses were wholly and accurately captured for data analysis. To achieve dependability, an audit trail was set up, which included clearly stating the research design and data collection process, as well as the steps taken to analyze the data. Confirmability was ensured by receiving feedback from the other research members in the team, who provided alternative perspectives and challenged any assumptions made by the corresponding researcher. The researchers compared their findings after they completed the analysis and reached a consensus after discussing their differences. In addition, experts in this research subject and in the area of qualitative research were asked for their opinions on each phase of the study. Lastly, rigor was ensured by using original quotes from the participants. In addition, the sample selection and data analysis process were explained in detail and findings were presented without any comments to ensure transferability [27].

## Results

The majority of the participants were female (90.0%) with a mean age of 44.6 years. The participants included four surgeons (20%), two anesthesiologists (10%), ten nurses (50%), and four physiotherapists (20%). An analysis of the data revealed eight main categories of perceived problems: (1) *patient selection*, (2) *referrals*, (3) *meeting the Health Care Guarantee*, (4) *patient flows*, (5) *homecare*, (6) *patient counseling*, (7) *transparency of the patient journey*, and (8) *receiving feedback*. In addition, problems related to information flow and communication, responsibilities between different stakeholders, and existing information systems were identified.

### Perceived problems related to pre-referral primary care

Problems during pre-referral primary care were related to patient selection (Table 2). According to the interviewees, primary care plays an insufficient role in the management of eligibility criteria before sending a referral. Healthcare professionals thought that a referral should not be sent until all indications and eligibility criteria for surgery are met and it would be fair to communicate the eligibility criteria to the patients on time to help them to meet the criteria before they come for specialist assessment. The following excerpt from one of the interviews expresses these views: “Sometimes we face these patients who have a body mass index over the agreed 35. We wish that the patients would realize that the targeted body mass index is not meant to harm them, but it is really a risk to have it so high…therefore they should find some way to reduce their weight before the surgery,” (interviewee no. 1, surgeon).

**Table 2:** Examples of perceived problems (*n* = 20)

	Main category	Generic category	Subcategory	Description
Phase 1	Problems in patient selection	Eligibility criteria	Late communication of eligibility criteria	Primary care plays an insufficient role in the management of (unbalanced) comorbidities and other eligibility criteria before sending a referral from primary care to a secondary care; referral should not be sent until all indications for surgery are met. Patients should also be informed earlier about eligibility criteria to motivate lifestyle changes (if appropriate).
			Lifestyle counseling	There are inconsistencies in municipalities organizing primary healthcare services and in coordinating lifestyle counseling. Patients’ attitudes to lifestyle counseling are variable.
Phase 2	Problems related to referrals	Incomplete referrals	Anamnestic information	Specialists are unable to comprehensively evaluate patients’ eligibility due to lack of anamnestic information (e.g., comorbidities, anticoagulants). In addition, there is a lack of physical performance indicators that would justify surgery.
			Radiographic examinations	Sometimes, imaging referrals or relevant radiographic examinations are lacking.
			Contact details	Patient’s (e.g., phone number, address), family’s (e.g., name, phone number, and address), and (if appropriate) health service’s (e.g., hospice care, in-home care) contact details are lacking.
		Contraindicated referrals	Processing of referrals	There are inconsistencies in sending back incomplete referrals.
			Standardized eligibility criteria	Patients are non-eligible for surgery, but they are still accepted regardless of (unbalanced) comorbidities, obesity, oral or skin health, physical performance, and/or smoking due to lack of standardized eligibility criteria/indications for surgery. There are non-eligible patients in the ImplantDB® register.
Phase 2–3	Problems to meet Health Care Guarantee	Scheduling	Complexity	Scheduling according to The Act on Specialized Health Care is a complex task due to non-eligible patients waiting for surgery, unnecessary appointments, cancelations, re-scheduling, and available resources. Availability of different types of appointments is not adjusted to needs (e.g., control visit, referral visit).
		Recourses	Staff capacity	Scheduling of specialist assessments and preoperative surgical visits is hampered due to lack of specialists. Correspondingly, scheduling of surgical procedures is hampered due to lack of scheduling times related to physicians’ shifts and vacations.
			Bed and room capacity	Scheduling of specialist assessments and preoperative surgical visits is hampered due to lack of rooms in outpatient clinics. Correspondingly, scheduling of surgical procedures is hampered due to lack of operating rooms and departmental closures (e.g., lack of post-surgery beds).
		Re-scheduling	Cancelations	Cancelations prior to preoperative surgical visits cause re-scheduling. The most frequent reasons for postponing surgery have been skin problems, medication, oral health, infections, drinking or eating, decreased health status, and/or anticoagulation.
Phase 3	Problems in patient flow	Preparation for surgery	Room capacity	Acute patient cases lead to a lack of free rooms. Elective patients need to wait for pre-surgery preparations and thus surgery.
			Documentation	Lack of electronic drug list causes double documentation prior to surgery. Surgery is documented several times. In addition, bone bank eligibility needs to be re-checked from hip-patients by filling in an interview form.
			Laboratory results	Lack of laboratory results causes delays and extra work prior the preoperative surgical visits and surgery.
		Preparation for discharge	Organization-related barriers	Organization-related implementation barriers (e.g., lack of commitment, placement, knowledge of discharge criteria), scheduling of daily rounds, and/or challenges in information transfer hinder the fulfillment of discharge criteria in a target time.
			Patient-related barriers	Patient-related barriers such as bleeding (e.g., hemoglobin mass alterations), lack of motivation, demobilization (e.g., range of motion, obesity), nausea, pain, and/or lack of escort hinder the fulfillment of discharge criteria in the target time.
Phase 4	Problems in homecare	Rehabilitation	Available services	There is a lack of services in rural areas, distances are too long, and resources to offer home-based physiotherapy are lacking. Generally, patients do not have the same rights.
			Patient’s compliance	Patient’s compliance with instructions is hampered due to lack of physical activity and motivation.
			Responsibilities between organizations	It is primary care’s responsibility to arrange post-surgery rehabilitation, but the control visit is back at the hospital. Possible follow-up visits and additional need for rehabilitation after the control visit are again organized under primary care.
			Information transfer	Information transfer from hospital to primary care is challenging, and thus, there can be mismatches in written instructions and lack of knowledge related to rehabilitation instructions given from the hospital.
			Early detection of problems	There is a limited possibility to detect problems with rehabilitation between discharge and control visits (e.g., range of motion, walking technique, leg length discrepancy).
		Recovery	Swelling	Patients have problems related to swelling and pain which hamper rehabilitation.
			Analgesia	Patients stop taking pain killers or pain killers and prescription run out too early. Unrelieved pain can result in chronic pain at a later date. Patients are afraid of drug dependence. Patients do not know how they could get new prescription.
Phase 1–4	Problems in patient counseling	Resources of counseling	Counseling time	Currently, there is less time than previously to counsel patients. This is caused by the optimization of the current journey prior and post-surgery.
			Number of patients	Currently, there is almost a double number of patients within the same time for pre-surgery visits.
			Counseling material	There is too much written counseling material prior to and post-surgery. Written materials do not include instructions about later life with a prosthetic joint. Paper-based questionnaires lead to double-documentation prior surgery. In addition, they are often returned empty or they have been wasted.
			Consistency of counseling	There are inconsistencies in counseling due to lack of understanding, hurry, job rotation, forgetfulness, and discrepancies in the instruction’s prior to surgery.
		Implementation of counseling	Timing of counseling	Counseling given too early (6 months prior surgery) or immediately post-surgery (with nausea and pain) which leads to the patient forgetting things. Currently, there is no time to change lifestyle due to late communication of eligibility criteria.
			Patient-centered counseling	Currently, written materials and permission forms are not personalized.
			Interaction during counseling	There is a lack of two-way communication prior to surgery. Patients are not ready to ask questions.
			Information overflow	All the information is provided in 30–60-min prior to surgery (without routine physiotherapist and anesthesiologist visits caused by optimization of the journey) and in 1.5 days post-surgery. Patients lose and forget essential information.
		Content of counseling	Counseling before admission	There is a lack of counseling related to bone bank, detailed information about the operation, management of recent changes in eligibility criteria (e.g., symptoms of flu or gastroenteritis, changes in medication, and/or skin problems), anesthesia and analgesia, as well as medicines, and natural remedies. In addition, patients need counseling related to complications even not of all wishes to hear about them. Current instructions of location, management of referrals, phases of the journey, and ward names are insufficient.
			Counseling during hospitalization	There is a lack of counseling related to detailed information about the time of the operation. Patients have worries related to their discharge on the second postoperative day. Patients do not know how and when they can go back home (e.g., sitting by car) and how to use aids. Patients need to understand what the target of the discharge is.
			Counseling before discharge	There is a lack of counseling related to recovery (e.g., swelling and wound care), expectations, and rehabilitation (e.g., how to exercise bike and for how long to use crutches). In addition, there is a lack of counseling related to pain management which causes limited physical exercises and thus reductions in the range of motion. Patients want to know whether their recovery is normal, better, or worse than others.
		Consequences of insufficient counseling	Patient’s preparation for a surgery	Patient’s inadequate preparation for surgery causes delays, extra work, and cancelations because familiarization with instructions is weak. In addition, walking aids and devices are not available and paper-based instructions are left at home/wasted.
			Numerous phone calls	There are a lot of phone calls related to the status of referrals, scheduling, timetables, and nature of the visit prior to surgery. In addition, there are a lot of calls to anesthesiologists related to eligibility criteria during pre-surgery visit. During homecare, there are a lot of contact-requests and re-calls (e.g., certificate of sick leave, lack of painkillers/prescription).
Phase 1–3	Problems in transparency	Unawareness of the patient journey	Confusion	Patients do not understand that all eligibility criteria need to be fulfilled prior to scheduling. Sometimes patients do not know, why they have received a referral. Naming of the wards, the reason for each appointment (e.g., specialist assessment, preoperative surgical visit, surgery), necessary examinations taken (e.g., laboratory results, imaging), and variations in waiting times and care givers causes confusion. Schedule (e.g., length of stay, daily rounds of the surgeon and physiotherapist) and actions taken (e.g., pain management, removal of urine catheter) post-surgery are unknown. Patients wonder when they can get pain killers and see the surgeon and physiotherapist.
Phase 1–4	Problems in receiving feedback	Written feedback	Targeted feedback	There is lack of written feedback or received feedback is published in newspapers. Feedback should be addressed to the right place. There is lack of positive feedback and subjective feedback of benefit to the surgery.

Abbreviations: *1* pre-referral primary care, *2* the specialist assessment and preoperative surgical visit, *3* in-hospital care, *4* homecare

### Perceived problems related to specialist assessment and the preoperative surgical visit

Problems during the specialist assessment and preoperative surgical visit were related to referrals and meeting the Health Care Guarantee (Table 2). Problems related to referrals were reported to be caused by incomplete and contraindicated referrals. Lack of anamnestic information, radiographic examinations, and reliable indicators of the patient’s physical performance makes it difficult to evaluate the eligibility of the patients. The following excerpt from of the interviews illustrates this view: “It is a challenge that we receive incomplete referrals, or that referrals are received but patients are not eligible for surgery. It means that with other illnesses that they have, the care balance is still missing, or that their BMI is too high , or they smoke or something else,” (interviewee no. 2, nurse).

The interviewees reported that the processing of referrals is not standardized and there is a lack of consistent eligibility criteria/indicators, or there appear to be at least some qualitative aspects in patient selection because patients were accepted regardless of unbalanced comorbidities and other risk factors. One of the interviewees expressed this problem by saying the following: “It (BMI) should be a mandatory field but is not in every referral. It depends on the person who reads the referral whether he or she bothers to send each of them back that are missing this aspect,” (interviewee no. 1, surgeon).

Meeting the Health Care Guarantee was found by the interviewees to be challenging in many ways. The interviewees pointed out that scheduling is a complex task because of contraindicated patients, unnecessary appointments, re-scheduling (due to canceled preoperative surgical visits and surgeries), and available resources (e.g., capacity constraints). One interviewee said: “Well, in practice the situation we have now is that we have shortage of resources at the outpatient clinic and so, meeting the, so called, Health Care Guarantee has been challenging,” (interviewee no. 3, surgeon). In addition, the availability of different types of appointments was thought not to be adjusted to the current need. The interviewees also reported a lack of information related to resources such as physicians’ shifts and vacations and department closures. Problems related to meeting the Health Care Guarantee are also mentioned in relation to *in-hospital care*.

### Perceived problems related to in-hospital care

The interviewees reported that problems during in-hospital care were related to patient-related workflow tasks (e.g., preparation for surgery and discharge) (Table 2). In addition, the interviewees noted that room capacity is very limited and sometimes elective patients need to wait for pre-surgery preparations and thus also wait for the actual surgery. For instance, two interviewees stated: “And then, we receive emergencies and their number can be unlimited. It depends on the numbers, for instance, if we get two joint replacement patients at 7 am in the morning, we do not necessarily have room for the other one, because other specialties are under so much pressure, and we have a limited number of patient beds. Then the patient has to wait at the lobby until 8 or 9 am until we will get a room,” (interviewee 4 and 5, both nurses). Also re-checking the bone bank eligibility in patient interviews and medications using paper-based questionnaires was mentioned by the interviewees as a cause of extra work and double documentation.

The interviewees stated that preparations for discharge were hampered due to organization (e.g., implementation and commitment of discharge criteria) and patient-related barriers (e.g., bleeding, nausea, pain, demobilization, lack of motivation, and escort). As one interviewee put it: “Overall it is challenging and I’m not quite sure about things, especially when residents change a lot. The physiotherapists are rotating too, and I have recently started wonder whether they are committed to our (process) idea that we discharge patients as soon as possible. They might quite easily leave it for the next day saying, ‘if you feel that way’. Sometimes I feel that they all are not committed to the idea,” (interviewee no. 6, nurse). In addition, the scheduling of daily rounds and challenges in information transfer were thought by the interviewees to hinder discharge within a targeted time.

### Perceived problems related to homecare

The interviewees thought there were problems during homecare and that these were often related to rehabilitation and recovery (Table 2). They also perceived a lack of services in rural areas and noted that long distances hinder their availability. The interviewees thought it was difficult to get physiotherapy at home, which violates the right of access to certain services regardless of the municipality of residence. For instance, one interviewee noted: “We have challenges with the patients that are living far away from the central areas. Sometimes patients need to take care of themselves if they cannot arrange any help. In some cases, physiotherapists have visited patients at home, but they cannot visit very often or stay for a very long time,” (interviewee no. 7, physiotherapist). In addition, it was thought by the interviewees that the prevailing practice does not support early detection of problems between discharge and control visits very well. For instance, some interviews felt there were difficulties related to the range of motion, walking technique, and leg length discrepancies which were not detected early enough.

The division of responsibilities and information transfer between organizations were reported by the interviewees to be challenging, and they mentioned that there could be mismatches between written instructions and a lack of knowledge related to rehabilitation instructions given from the hospital. In addition, it was felt that some patients are not necessarily compliant to rehabilitation and they suffer a lack of motivation. One major issue hindering recovery is pain management at home. Swelling and pain make rehabilitation more difficult, but the interviewees noted that some patients do not take pain killers long enough or that their prescription may run out too early. It was also noted that some patients were afraid of drug dependency or did not know how to renew their prescriptions.

### Perceived problems related to general process

Problems that were not directly linked to any of the four phases were categorized as “general process problems.” These issues, raised by the interviewees, were related to patient counseling, transparency of the journey, and receiving feedback (Table 2). The interviewees reported that the quality of counseling had changed due to the fast-track methodology. Currently, it was felt in the interviews that there was less time for counseling due to organizational optimization. Also, the number of patients to be counseled had been doubled. One of the physiotherapists in the interviews noted: “We have used the new fast-track care protocols and now we have a new challenge related to the scheduling. Previously, we gave the same counselling over three days that we now do in one day. That means that we have more patients in a shorter amount of time. Often, the patient may have pain and they might feel bad when we give them the guidelines. The next day, they may even think that have not even seen the physiotherapist,” (interviewee no. 8, physiotherapist). In addition, it was reported in some of the interviews that patients were experiencing information overload because all of the information is provided in 30–60 min prior to surgery and in 1.5 days post-surgery. The interviewees felt that some patients could easily lose or forget some essential information.

The interviewees also mentioned some problems with counseling, which they thought was not 100% consistent because there are differences in how well patients are able to receive the information. They also mentioned that the personnel may be in a hurry, or not familiar with practices because of job rotation, or that they might forget to carry out practices. The interviewees also said there were sometimes discrepancies between written instructions prior to surgery. According to the healthcare professionals, there is also lack of two-way communication, because patients are not ready to ask questions immediately after receiving the information. The following excerpt illustrates some typical thoughts on these issues: “So the material is in one big “bundle” on the day of discharge. Sometimes I have given a terrible monolog, and then when I ask: ‘Do you have any questions?’ they might be stunned and say things like : ‘Well, I don’t have any really,’ and then they may come later. I would think that they would have questions later,” (interviewee no. 6, nurse).

Several problems were identified related to the content of counseling prior to, during, and post-surgery (Table 2). The interviewees said that patients also ask how to deal with recent changes in their health status that could influence their eligibility for surgery, as for instance in this short extract from one of the interviews: “It could be something such as if they have the flu coming on, or then a stomach bug, they might ask if they are eligible for surgery. Or it might be about the medication,” (interviewees 4 and 5, both nurses). In addition, the interviewees said that patients would like to know whether their recovery is normal, better, or worse than others, but that healthcare professionals find it difficult to give such estimations.

#### Transparency

The interviewed healthcare professionals thought it was problematic that patients do not necessarily know what to expect when they come to hospital and what will happen during the care journey. The interviewees thought that patients did not necessarily know why they had received referral for specialized medical care or that they need to be eligible for surgery before the surgery is scheduled. The naming of the wards, the necessary examinations to be taken, and the reason for each appointment were reported to cause confusion. One interviewee described this by saying: “They might often ask whether this is the surgery appointment. So clearly, in these case, they don’t understand that the process that comes first is the pre-operative surgical visit. And then, if you meet all the criteria, you will go to surgery. And then they don’t understand that there should be a checklist including weight, teeth, basic illnesses, skin, all of these. When these aspects are all checked, then the referral would come,” (interviewee no. 2, nurse). In addition, the interviewees noted that patients were not familiar with the care schedules, such as varying waiting times, a typical length of stay, and the daily rounds of surgeons and physiotherapists. They also mentioned that changes in care givers can be confusing. Furthermore, it was reported that actions taken post-surgery, such as pain management and removal of urine catheters, were often unknown to the patients.

#### Receiving feedback

The interviewed healthcare professionals felt that they do not get enough written feedback from the patients. This is collected after a control visit, but it is often not addressed to the right ward or place during the care journey. The interviewees also felt that the feedback given directly to healthcare professionals was often positive, but written feedback is typically negative. Sometimes, unhappy patients do not give the feedback directly to the hospital, but their experiences are published in letters to an editorial section of a newspaper. Through the feedback that is collected, the interviewees felt it was not possible to get subjective feedback on the benefits of the surgery itself. One surgeon expressed this by saying: “We are interested in the results of the surgery. We are interested to know whether the surgery was beneficial for patients, what kinds of experiences the patients have had at different phases, and whether the care was good or not. We are interested to know how to improve the care. Currently we are not getting this type of feedback from the patients,” (interviewee no. 3, surgeon).

## Discussion

The findings from this prospective study provide a rich description of the experiences of healthcare providers of the implemented fast-track methodology. The analysis of the data revealed eight main categories of problems: patient selection, referrals, meeting the Health Care Guarantee, patient flows, homecare, patient counseling, transparency of the care journey, and receiving feedback. In addition, problems related to information flows and communication, responsibilities between different stakeholders, and the existing information systems were identified.

The detected problems reduce the effectiveness of the care journey and make it more difficult to meet the Health Care Guarantee. Most of the perceived problems (e.g., patient selection, referrals, waiting times and waiting list management, counseling resources, preparation for discharge, and responsibilities between different stakeholders) are related to both internal and external organizational practices and, thus, can be solved by organizational and/or managerial changes. Part of the revealed problems (e.g., preparation for surgery, counseling implementation and content, information flows and communication, transparency, and receiving feedback) could be alleviated by utilizing information and communication technologies [13–18, 20].

Problems regarding patient selection were observed between referring physicians and specialists. In the previous literature, orthopedic surgeons have applied less-stringent criteria than referring physicians while rest pain, pain with activity, and functional limitations have been the most important indications for THA [28]. The observed differences between these stakeholders can lead to variations and perhaps inequities in the provision of care.

Similar problems regarding a lack of anamnestic information of presurgical risk factors and reliable indicators of a patient’s physical performance caused problems in patient selection. According to the previous literature, presurgical risk screening tools are needed to predict surgical outcomes, to identify factors impacting healthcare service delivery and/or costs, and to predict discharge planning requirements [29].

Detected problems in patient selection and referrals in conjunction with capacity constraints make it difficult to meet the Finnish Health Care Guarantee. Patient selection and referral processing are complex tasks where professionals have to often make case by case decisions and search for information to support them. In the previous literature, the main barriers that have been hampered waiting lists and waiting time management have been organizational (e.g., physician involvement, human resource capacity, and information management systems) and contextual (e.g., stakeholders’ engagement, funding) factors [8].

Generally, in the related literature, fast-track THA/TKA has been a feasible method for most of the patients. However, for patients over 80 years old, preoperative cardiopulmonary diseases, preoperative use of mobility aids, and living conditions have been associated with delayed discharges, whereas the readmission rates have not differed between older or younger patients, or those with or without cardiopulmonary diseases, or nothing to do with the use of mobility aids [29–31]. In this study, preparation for discharge was hampered due to organization- (e.g., implementation and commitment of discharge criteria) and patient-related barriers (e.g., bleeding, nausea, pain, demobilization, lack of motivation and escort). In the previous literature, delayed discharge has mostly been related to medical, social, and organizational reasons [29, 30].

The advantages of patient counseling are well-documented from both socio-economic [32, 33] and patient perspectives [34, 35]. According to the participants of this study, the implementation of individual, oral, and multidisciplinary counseling in conjunction with written material was not considered patient-centered or interactive due to the lack of timing optimization and patient-specific needs taken into account when planning counseling, or while goal-setting prior to and post-surgery. In addition, the experiences describe the current situation very well where patients still are objects of care instead of being active subjects. According to Berend et al. [3], it would be beneficial to provide written and electronic patient educational materials, videos, and educational lessons for patients and their family when the schedule is initially scheduled. Moreover, future efforts to enhance recovery and reduce the length of hospital stays should be focused on analgesia, prevention of orthostatism, and rapid recovery of muscle function [36]: lack of or inadequate counseling related to pain management can reduce the performance of physical exercises and thus lead to reductions in the range of motion post-surgery. In addition, unrelieved pain can result in chronic pain at a later date [37].

Detected problems with areas of responsibility were observed between and within organizations. Due to organizational consequences related to nursing care for fast-track patients, nurses have inherited some tasks from surgeons and physiotherapists, and thus gained more responsibility (an expanded and enhanced role). This has occurred, for instance, in pain management and mobilization, which is in line with Specht et al. [7] who have earlier pointed this trend out. Although the study was conducted in specialized medical care, problems were also identified prior and post-surgery where the responsibilities were shared with primary care. In order to remove these problems and improve the patient flow through the entire care journey, all stakeholders who provide care during the care journey should be involved in the development work. Development can be started from an individual organization, but big problems remain unsolved if the care journey is not considered as a whole. What can be done is to disseminate the study results and suggest new roles and responsibilities for organizations that are in charge of the healthcare renewal.

Detected problems in existing information systems, the information flow, and communication were related to lack of structured referral, lack of integration between medical and dental records as well as radiographic and other image-based data, and lack of (continuous) electronic feedback. In addition, detected problems in the information flow and communication hamper the transparency of the care journey.

Our study has several limitations. Firstly, this study shows the specific care journey in a very negative light, but actually its quality has been recently awarded. The purpose of this study was to gain a thorough understanding of problems to be solved for future improvements. Because the research approach was qualitative, all problems have been reported, even if they have occurred only once in the data. Secondly, the interviews were conducted within a single hospital: for this reason, organizational policies or aspects of organizational culture that are unique to this organization may not reflect experiences in other nursing work environments. However, many of the themes reported and identified in the current work align with the prior literature. Thirdly, the topic guide was not pilot-tested. Finally, the transcripts were not returned to the participants for comment or correction. However, because the transcripts were transcribed verbatim from the recordings, they can be considered reliable sources of information of the experiences of the healthcare personnel.

In the future, organizational culture (e.g., shared ways of thinking, feeling, and behaving) and theories could be utilized to explain how internal and external practices are formed and how they can be changed to alleviate problems during the surgery care journey [38]. In addition, more research could be done focusing on the managerial practices, including staff satisfaction, related to the improvements made in the joint replacement center units. Novel information and communication technologies are needed for organizational optimization to result in a streamlined care journey, better access to healthcare services, and improved outcomes and to take the patient experience to the next level. New technological solutions could provide up-to-date communication channels between the healthcare personnel and the patients and support the patient during the care journey in a more interactive way compared to paper-based instructions. Technology could also be developed to support patient selection. In addition, the patient’s active participation in the process is also needed.

## Conclusion

The study revealed that healthcare professionals perceived several problems during the fast-track journey that reduce its effectiveness and make more difficult to meet the Finnish Health Care Guarantee. Problems could be alleviated by changing internal and external organizational practices, as well as by developing new information and communication technologies that would provide up-to-date communication channels for healthcare professionals and patients. In addition, new collaboration mechanisms should be developed in order to solve the problems that go across different organizations.

## Data Availability

The datasets generated and analyzed are not publicly available because ethical and legal restrictions related to the confidentiality of study participants prohibit publicly available datasets.

## Abbreviations

THA: Total hip arthroplasty
TKA: Total knee arthroplasty
